# Hypoadrenocorticism in dogs under UK primary veterinary care: frequency, clinical approaches and risk factors

**DOI:** 10.1111/jsap.13285

**Published:** 2021-02-08

**Authors:** I. Schofield, V. Woolhead, A. Johnson, D. C. Brodbelt, D. B. Church, D. G. O'Neill

**Affiliations:** ^1^ Pathobiology and Population Sciences The Royal Veterinary College Hawkshead Lane, North Mymms, Hatfield Herts AL9 7TA UK; ^2^ Eastcott Referrals, Edison Park Dorcan Way, Swindon SN3 3RB UK; ^3^ Clinical Science and Services, The Royal Veterinary College Hawkshead Lane, North Mymms, Hatfield Herts AL9 7TA UK

## Abstract

**Objectives:**

To estimate the frequency, clinical approaches and risk factors of hypoadrenocorticism in dogs under UK primary veterinary care.

**Materials and Methods:**

Dogs diagnosed with hypoadrenocorticism were identified from the UK VetCompass™ programme by searching anonymised electronic patient records. Pre‐existing and newly diagnosed cases of disease during 2016 were included. Cases were further sub‐categorised as having a laboratory‐confirmed or presumed diagnosis of hypoadrenocorticism based on the information recorded in the electronic patient records. Descriptive data were manually extracted. Multivariable logistic regression methods were used to identify demographic risk factors.

**Results:**

There were 177 hypoadrenocorticism cases identified from 905,543 dogs in 2016; 72 laboratory‐confirmed and 105 presumed. The 1‐year period prevalence for hypoadrenocorticism in all dogs was 0.06% (95% confidence interval: 0.05‐0.07%). The most common presenting clinical signs in laboratory‐confirmed dogs were lethargy (51/66, 77.3%), anorexia (48/66, 66.7%) and vomiting (48/66, 66.7%). Hyperkalaemia was reported in 47 of 53 (88.7%), hyponatraemia in 46 of 53 (86.8%). Median sodium: potassium ratio was 19.00 (interquartile range: 16.20‐20.60). Breed, age, neuter status and insurance status were associated with a laboratory‐confirmed diagnosis of hypoadrenocorticism. No sex association with hypoadrenocorticism was observed in the multivariable model. The standard poodle had 51.38 times the odds (95% CI: 14.49‐182.18) of hypoadrenocorticism compared with crossbreeds. The labradoodle and West Highland white terrier also had increased odds.

**Clinical Significance:**

This is the first epidemiological study to report on hypoadrenocorticism in dogs within the UK primary‐care population. These results provide benchmark data of current veterinary activity relating to hypoadrenocorticism in primary‐care practices.

## INTRODUCTION

Hypoadrenocorticism (Addison's disease) is an uncommon but clinically important disease in dogs due to the potential progression to a critical emergency presentation, the “Addisonian crisis” (Feldman & Nelson 2004). Hypoadrenocorticism most commonly presents following primary adrenocortical disease which results in a significant reduction in the secretion of mineralocorticoids and glucocorticoids from the adrenal cortex (Peterson *et al*. 1996, Feldman & Nelson 2004). Primary hypoadrenocorticism most frequently results following immune‐mediated destruction of the adrenal cortex, although multiple aetiologies are reported in the literature (Boujon *et al*. 1994, Feldman & Nelson 2004, Adissu *et al*. 2010, Kook *et al*. 2010, Frank *et al*. 2013). Typical and atypical forms of primary hypoadrenocorticism are reported however the definition of atypical hypoadrenocorticism is currently ongoing with differing definitions included within the literature (Peterson *et al*. 1996, Thompson *et al*. 2007, Baumstark *et al*. 2014, Wakayama *et al*. 2017). Secondary hypoadrenocorticism results from pituitary disease causing a deficiency of adrenocorticotropic hormone (ACTH), leading to a glucocorticoid insufficiency (Peterson *et al*. 1996, Kintzer & Peterson 1997).

The frequency of hypoadrenocorticism in dogs has been rarely reported. Based on an insured population of Swedish dogs, the prevalence was estimated at 0.09% and the incidence rate was 2.26 cases/1000 dog years at risk (Hanson *et al*. 2016). To date, no studies have reported the frequency of hypoadrenocorticism for dogs in the UK.

Dogs with hypoadrenocorticism generally present with varying and non‐specific clinical signs including vomiting, lethargy, weight loss, diarrhoea and inappetence, and therefore the disease is considered challenging to diagnose as it is often overlooked as a differential diagnosis (Peterson *et al*. 1996). Clinicopathological findings such as hyperkalaemia, hyponatraemia and a reverse or absent stress leukogram can increase suspicion of hypoadrenocorticism (Adler *et al*. 2007, Seth *et al*. 2011, Reagan *et al*. 2019). Some risk factors associated with hypoadrenocorticism in dogs have been described. Cases are reported to first present at around 2‐6 years of age (Peterson *et al*. 1996, Feldman & Nelson 2004). Breeds including the Portuguese water dog, standard poodle, cocker spaniel, bearded collie, Cairn terrier, wheaten terrier, West Highland white terrier and rottweiler have been reported with increased risk of hypoadrenocorticism (Peterson *et al*. 1996, Oberbauer *et al*. 2002, Famula *et al*. 2003, Hanson *et al*. 2016). Females were reported at over twice the risk of hypoadrenocorticism in an older study in the USA (Peterson *et al*. 1996). A sex association is not consistently reported in other studies (Oberbauer *et al*. 2002, Famula *et al*. 2003).

The current study had three main objectives: (1) to estimate the frequency of hypoadrenocorticism in dogs under primary veterinary care in the UK; (2) to describe the diagnosis and clinical management of hypoadrenocorticism and (3) to report risk factors for hypoadrenocorticism. It was hypothesised that females have an increased odds of hypoadrenocorticism compared to males.

## METHODS

A cross‐sectional analysis of a retrospective cohort was used to report the frequency and to examine the risk factors for dogs having hypoadrenocorticism. The study used routinely recorded primary‐care clinical data collected within VetCompass™ in the UK (VetCompass 2020) and included all dogs under veterinary care at collaborating practices in 2016. Dogs under veterinary care were defined as having either (1) at least one electronic patient record (EPR) documented during 2016 or (2) at least one EPR documented during 2015 and 2017. Available clinical data for the study included the demography, free text clinical notes and prescribed treatments recorded within the anonymised EPRs. Search terms were applied to the study population to identify candidate dogs with increased probability of hypoadrenocorticism: “Addis*, hypoadreno*, hypoa, zycort*, DOCP, florinef, fludro*.” Dogs were eligible for inclusion as a hypoadrenocorticism case if a pre‐existing (first diagnosed before 2016) or incident (first diagnosed within 2016) diagnosis of hypoadrenocorticism was recorded within the EPR between January 1, 2016 and December 31, 2016. Cases were further sub‐categorised into a “laboratory‐confirmed” group (evidence of a supportive diagnostic ACTH stimulation test recorded by the veterinarian within the clinical free text) and a “presumed” group (an ACTH stimulation test was not recorded within the clinical records). Dogs with a diagnosis of iatrogenic hypoadrenocorticism recorded within their EPRs or glucocorticoids (topical or systemic) prescribed within 30 days before an ACTH stimulation test being performed were excluded. The full clinical records of a random selection of candidate dogs, based on the sample size estimation, were manually checked to evaluate against the hypoadrenocorticism case inclusion criteria. All dogs that were not identified by the search terms as candidate cases during the initial screening were included as non‐cases for hypoadrenocorticism in the risk factor analysis.

**FIG 1:**
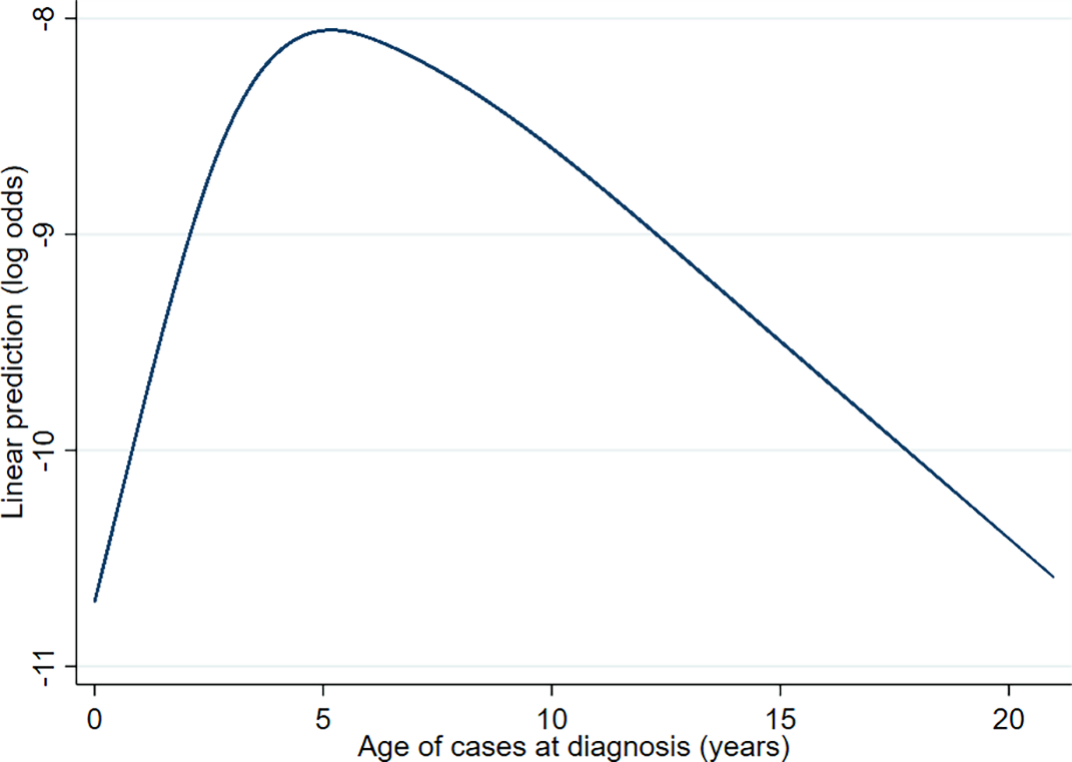
Non‐linear association of the age of dogs diagnosed with hypoadrenocorticism under primary veterinary care in the UK (n = 72)

Available demographic data for study dogs included date of birth, sex, neuter status, insurance status, breed, mean lifetime bodyweight above 18 months and veterinary clinic identification. Continuous variables were assessed for linearity with the outcome; where the associations were non‐linear, they were categorised for analysis (Dohoo *et al*. 2012). Age (years) was calculated using the date of birth and the date of initial diagnosis for cases, or at the end of the study period (December 31, 2016) for non‐cases. Age was categorised into three groups based on graphical assessment of the non‐linearity: <4, 4 to <9 and ≥9 (years) (Fig 1) (Harrell Jr 2015). Individual purebred and designer breeds were included in the risk factor analysis if there were ≥5 hypoadrenocorticism case dogs of that breed. All other breeds with <5 cases were grouped as either; (1) “purebred other” if they were a registered purebreed (VeNom Coding Group 2018), or (2) “crossbred” if they were recorded as a crossbreed or a designer breed cross (*e.g*. pug × beagle or puggle). Bodyweight (kg) described the mean from all bodyweight data recorded for dogs aged over 18 months and was split into four categories: <10, 10 to <20, 20 to <30 and ≥30. Neuter and insurance status was recorded at the end of the study period for cases and non‐cases. Additional descriptive data were extracted by manual revision of the case EPRs, including date of first diagnosis, clinical signs at presentation, laboratory tests used at diagnosis and treatment data. Cases were classified as either pre‐existing (before 2016), incident (during 2016) or unknown based, on the date of diagnosis recorded within the EPRs.

Data were cleaned in Excel (Microsoft Corp.) and uploaded into Stata 15 (Stata, TX, USA) for statistical analysis. Categorical data were presented showing the count and corresponding percentage. Quantitative data were assessed for normality with Shapiro‐Wilk tests and graphically; normally distributed data were summarised using the mean [standard deviation (sd)] and non‐normally distributed data using the median [interquartile range (IQR) and range]. Sample size calculations estimated a cross‐sectional study with 157,000 male and 157,000 female animals for a disease with a 0.09% prevalence (Hanson *et al*. 2016) would have 80% power to detect sex as a risk factor with an odds ratio (OR) of 1.5 (OpenEpi 2018).

To estimate the 1‐year period prevalence and incidence risk of hypoadrenocorticism in 2016 within dogs under veterinary care, the manually verified case counts were weighted by the inverse of the proportion of candidate cases to take into account the sampling design of the study (O'Neill *et al*. 2016). The estimated total hypoadrenocorticism case count was used to calculate overall and breed prevalence values based on a denominator of all study dogs either overall or by breed, respectively. The confidence interval (CI) estimates were derived from standard errors, based on approximation to the binomial distribution (Kirkwood & Sterne 2010).

Univariable and multivariable binary regression modelling were used to assess associations between risk factors and hypoadrenocorticism, with clinic ID included as a random effect. Analyses were performed for laboratory‐confirmed cases only. Risk factors with a broad association with the outcome during univariable analysis (likelihood ratio test (LRT) = P < 0.2) were considered for multivariable evaluation. Multivariable model building used a forwards stepwise manual approach. To address the study hypothesis, sex was included a priori in the multivariable model. Confounding effects were assessed by observing for a >10% change in the ORs following inclusion of an additional risk factor (Katz 2011). Clinic ID was included as a random effect to account for the correlated nature of the data across different veterinary clinics. Linearity of age effects on the outcome was assessed by graphical assessment of the log odds of the outcome and testing for a departure from a linear trend using a LRT (Clayton & Hills 2013). Biologically plausible pairwise interactions in the final model were examined with the LRT of homogeneity (Dohoo *et al*. 2012). Statistical significance was set at P < 0.05. Goodness of fit and discrimination of the final model was evaluated by the Hosmer‐Lemeshow test and the area under the receiver operating characteristic (ROC) curve (Hosmer Jr *et al*. 2013, O'Neill *et al*. 2020). Ethical approval was granted by the RVC Ethics and Welfare Committee (SR2018‐1652).

## RESULTS

The study population included 905,543 dogs under primary veterinary care across 886 VetCompass™ participating practices in 2016. Search terms identified 9712 candidate dogs with evidence of consideration of hypoadrenocorticism in their EPRs. The EPRs of 3000 (30.8%) randomly selected dogs were manually examined and identified 184 cases of hypoadrenocorticism in 2016. However, seven dogs had been prescribed glucocorticoids in the 30 days before performing an ACTH stimulation test and were excluded from further analysis. Of the remaining 177 dogs, 72 (40.7%) had a laboratory‐confirmed diagnosis and 105 (59.3%) had a presumed diagnosis of hypoadrenocorticism. The reasons for classifying the diagnosis as presumed in 105 include 17 (16.2%) dogs with a prior diagnosis made at a referral or emergency veterinary practice, 77 (73.3%) with a prior diagnosis at another veterinary practice (type of practice unknown) and 11 (10.5%) with the type of diagnostic test performed as unrecorded. Of the 177 recorded cases, 33 (18.6%) were first diagnosed in 2016 (incident cases), 120 (67.8%) were diagnosed before 2016 and the date of diagnosis was unavailable in 24 (13.6%) dogs. There were 26 of 177 (14.7%) cases recorded as primary hypoadrenocorticism and the remaining 151 were not explicitly differentiated; no cases were specifically recorded with secondary hypoadrenocorticism. Additionally, 10 of 177 (5.6%) cases were recorded as atypical within the EPRs.

Median age at initial diagnosis for laboratory‐confirmed cases was 5.18 years (n = 72, IQR: 3.15‐6.79; range: 0.14‐13.39). Median age of non‐cases on the December 31, 2016 was 4.42 years (n = 883,436; IQR: 1.86‐8.05; range: 0.003‐20.97). Median bodyweight for laboratory‐confirmed cases was 19.58 kg (n = 67; IQR: 10.14‐28.77; range: 3.72‐68.88) and for non‐cases was 13.96 kg (n = 586,481; IQR: 8.18‐25.00; range: 0.72‐90.30). Of the 72 laboratory‐confirmed cases, 31 (43.1%) were female and 41 (56.9%) were male. For non‐cases 427,019 (47.9%) were female and 464,593 (52.1%) were male. The most represented breeds with laboratory‐confirmed hypoadrenocorticism were the West Highland white terrier (n = 6; 8.3%), labradoodle (4; 5.6%), standard poodle (4, 5.6%) and the cocker spaniel (n = 4; 5.6%). Comparison between laboratory‐confirmed *versus* presumed cases showed comparable demographics (Table 1). There was a greater proportion of missing data for the presumed cases than for the laboratory‐confirmed cases.

**Table 1:** Descriptive statistics of risk factors for hypoadrenocorticism in dogs attending UK first opinion practice in 2016 within the VetCompass programme, comparing laboratory‐confirmed cases (evidence of a supportive diagnostic ACTH stimulation test recorded by the veterinarian within the clinical records) and presumed cases (either an ACTH stimulation test was not recorded within the clinical records or glucocorticoids (topical or systemic) were prescribed within 30 days before an ACTH stimulation test being performed). (Cases n = 177; laboratory‐confirmed cases n = 72; presumed cases n = 105; non‐cases n = 895,831)

	Variable	All cases (%)	Laboratory‐confirmed cases (%)	Presumed cases (%)	Non‐case (%)	P‐value confirmed *versus* presumed^1^
Age (years)	<4	46 (26.0)	21 (29.2)	25 (23.8)	410,389 (45.8)	<0.001
	4‐ < 9	84 (47.5)	45 (62.5)	39 (37.1)	298,182 (33.3)	
	≥9	23 (13.0)	6 (8.3)	17 (16.2)	174,865 (19.5)	
	Unknown	24 (13.6)	0 (0.0)	24 (22.9)	12,395 (1.4)	
Weight (kg)	<10	40 (26.9)	15 (22.4)	25 (30.5)	211,967 (36.1)	0.669
	10‐ < 20	40 (26.9)	19 (28.4)	21 (25.6)	165,214 (28.2)	
	20‐ < 30	38 (25.5)	17 (25.4)	21 (25.6)	114,595 (19.5)	
	≥30	31 (20.8)	16 (23.9)	15 (18.3)	94,705 (16.2)	
Sex	Female	89 (50.6)	31 (43.1)	58 (55.8)	427,019 (47.9)	0.097
	Male	87 (49.4)	41 (56.9)	46 (44.2)	464,593 (52.1)	
Neuter status	Entire	60 (34.1)	19 (26.4)	41 (39.4)	489,820 (54.9)	0.073
	Neutered	116 (65.9)	53 (73.6)	63 (60.6)	401,792 (45.1)	
Insurance	Uninsured	117 (66.1)	45 (62.5)	72 (68.6)	781,706 (87.3)	0.402
	Insured	60 (33.9)	27 (37.5)	33 (31.4)	114,125 (12.7)	
Breed	Crossbreed	36 (20.3)	13 (18.1)	23 (21.9)	234,318 (26.2)	0.715
	Purebreed other	72 (40.7)	31 (43.1)	41 (39.0)	464,335 (51.8)	
	Border collie	5 (2.8)	2 (2.8)	3 (2.9)	22,167 (2.5)	
	Cocker spaniel	8 (4.5)	4 (5.6)	4 (3.8)	31,787 (3.6)	
	Jack Russell terrier	12 (6.8)	3 (4.2)	9 (8.6)	47,935 (5.4)	
	Labradoodle	11 (6.2)	4 (5.6)	7 (6.7)	7424 (0.8)	
	Labrador retriever	12 (6.8)	3 (4.2)	9 (8.6)	59,268 (6.6)	
	Standard poodle	8 (4.5)	4 (5.6)	4 (3.8)	1057 (0.1)	
	West Highland white terrier	9 (5.1)	6 (8.3)	3 (2.9)	18,443 (2.1)	

^1^ Chi‐squared P‐value

### Disease frequency

After accounting for the effects of the subsampling protocol, the estimated 1‐year period prevalence for hypoadrenocorticism in dogs in 2016 was 0.06% (95% CI: 0.05‐0.07%) and the 1‐year incidence risk in 2016 was 0.01% (95% CI: 0.01‐0.02%). When including laboratory‐confirmed cases only, the 1‐year period prevalence was 0.03% (95% CI: 0.02‐0.04%) and the 1‐year incidence risk was 0.008% (95% CI: 0.005‐0.01). The standard poodle had the highest breed prevalence, with hypoadrenocorticism recorded in 2.26% of the breed (95% CI: 1.14‐4.43%) followed by the labradoodle (0.47%, 95% CI: 0.26‐0.84%) and the West Highland white terrier (0.15%, 95% CI: 0.08‐0.30) (Table 2).

**Table 2:** Overall estimated breed prevalence of hypoadrenocorticism in dogs under primary veterinary care in the UK (all cases n = 177; laboratory‐confirmed cases n = 72; population n = 905,543)

Breed	Prevalence all cases (%)	95% confidence interval	Prevalence laboratory‐confirmed cases (%)	95% confidence interval
Standard poodle	2.26	1.14‐4.43	1.14	0.43‐3.00
Labradoodle	0.47	0.26‐0.84	0.17	0.06‐0.45
West Highland white terrier	0.15	0.08‐0.30	0.10	0.05‐0.23
Cocker spaniel	0.08	0.04‐0.16	0.04	0.02‐0.11
Jack Russell terrier	0.08	0.05‐0.14	0.02	0.01‐0.06
Border collie	0.07	0.03‐0.17	0.02	0.01‐0.12
Labrador retriever	0.07	0.04‐0.11	0.02	0.01‐0.05
Crossbreed	0.05	0.03‐0.07	0.02	0.01‐0.03
Purebreed other	0.05	0.04‐0.06	0.02	0.02‐0.03

### Diagnosis and clinical management

**FIG 2:**
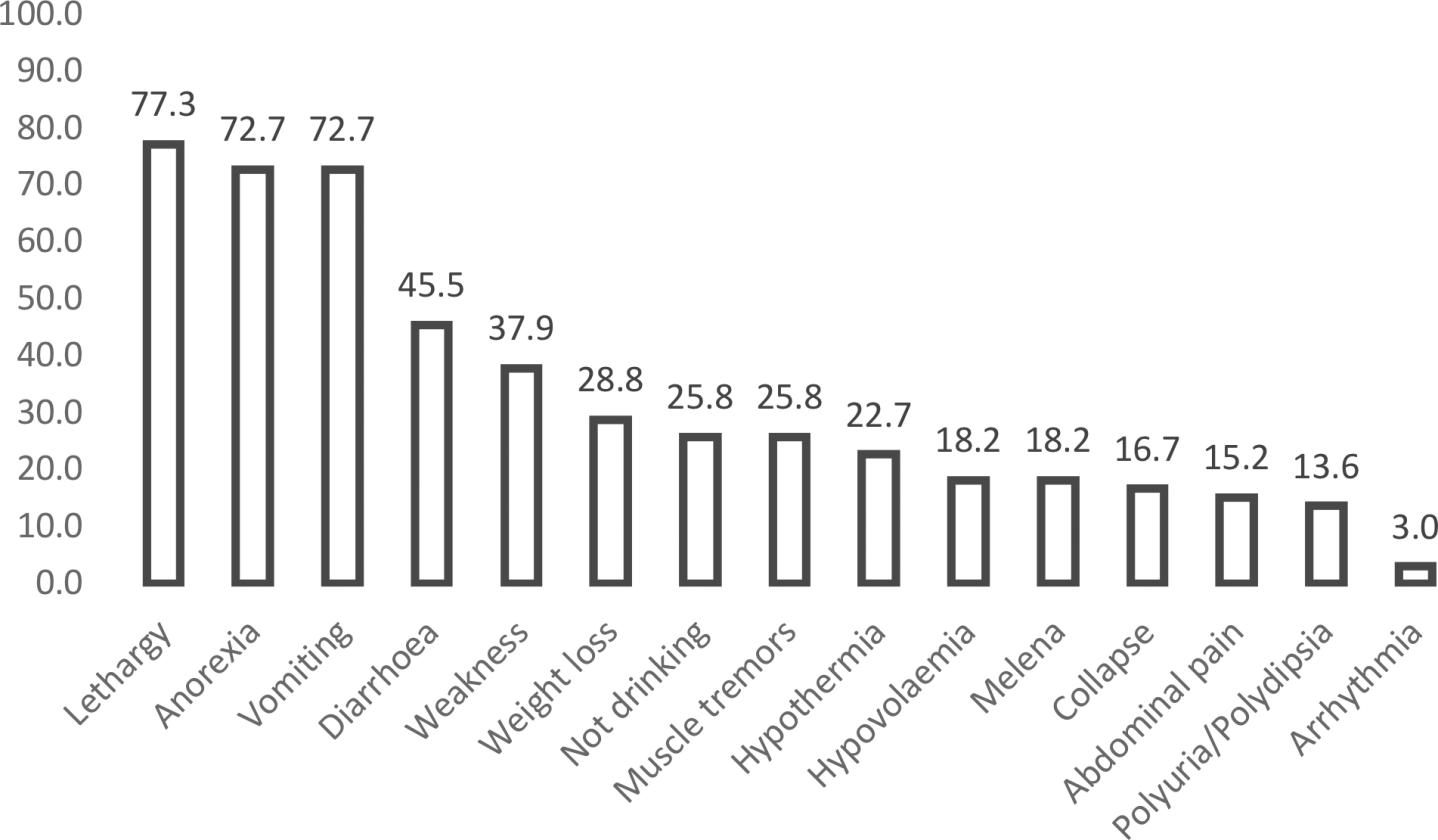
Presenting clinical signs at the time of diagnosis in dogs with hypoadrenocorticism under primary veterinary care in the UK (n = 66)

Diagnostic and clinical management data were reported for laboratory‐confirmed cases only. Clinical signs at presentation were recorded for 66 of 72 dogs with a laboratory‐confirmed diagnosis. The most commonly recorded clinical signs were lethargy (51/66, 77.3%), anorexia (48, 72.7%), vomiting (48, 72.7%), diarrhoea (30, 45.5%), weakness (25, 37.9%) and weight loss (19, 28.8%) (Fig 2). Potassium and sodium concentrations at diagnosis were recorded for 53 of 72 (73.6%) dogs with a laboratory‐confirmed diagnosis. Hyperkalaemia was reported in 47 of 53 (88.7%) and hyponatraemia was reported in 46 of 53 (86.8%), according to the reference ranges reported. Median sodium: potassium ratio was 19.00 (n = 38, IQR: 16.20‐20.60, range: 12.90‐26.00).

All laboratory‐confirmed cases had an ACTH simulation test performed. In addition to this, endogenous aldosterone concentrations were measured in 3 of 72 dogs (4.2%). Measurement of endogenous ACTH concentration was not recorded in any dog. Four of the presumed cases had evidence that a basal cortisol concentration had been measured.

In dogs with a laboratory‐confirmed diagnosis of hypoadrenocorticism, the most frequently administered initial treatments included intravenous fluid therapy (IVFT) (n = 55/72, 76.4%), gastric protectants (50/72, 69.4%), injectable dexamethasone (44/72, 61.1%), prednisolone (41/72, 56.9%) and antibiotics (26/72, 36.1%). The longer‐term clinical management protocols as recorded by the end of the study period were: desoxycorticosterone pivalate (DOCP) + prednisolone (n = 49/72, 68.1%), fludrocortisone + prednisolone (9/72, 12.5%), DOCP, cortisone + prednisolone (5/72, 6.9%), fludrocortisone only (4/72, 5.6%), prednisolone only (3/72, 4.2%) and fludrocortisone + prednisolone + cortisone (2/72, 2.8%). Mean starting injection of DOCP was 2.15 mg/kg (n = 35, sd: 0.19) and was given at a mean interval of 29 days (n = 44, 15.32±). Median duration of treatment with DOCP was 208 days (IQR: 168‐240; range: 10‐263). Mean starting dose of fludrocortisone was recorded as 0.01 mg/kg/day (n = 46, 0.01±) and was most frequently administered once daily (n = 30/46, 65.2%). Median duration of treatment with fludrocortisone was 1471 days (IQR: 1073‐1910; range 212‐2848). Mean prednisolone dose was 0.26 mg/kg/day when given with DOCP (n = 33, 0.15±) and 0.29 mg/kg/day when given with fludrocortisone (n = 8, 0.16±).

### Risk factor analysis

Univariable analysis identified neuter status, age, breed, bodyweight and insurance status as broadly associated with dogs having hypoadrenocorticism (P < 0.20) (Table 3). Bodyweight was not taken forward for multivariable modelling because it was considered collinear with breed.

**Table 3:** Univariable analysis of risk factors for hypoadrenocorticism in dogs attending UK primary‐care practice in 2016 within the VetCompass programme, for laboratory‐confirmed only. Veterinary clinic was included as a random effect (laboratory‐confirmed cases n = 72; non‐cases n = 895,831)

	Variable	Odds ratio	95% Confidence interval	P‐value^1^
Age (years)	<4	Baseline	–	–
	4‐ < 9	2.88	1.71‐4.84	<0.001
	≥9	0.64	0.26‐1.60	0.342
Weight (kg)	<10	Baseline	–	–
	10‐ < 20	1.62	0.82‐3.20	0.161
	20‐ < 30	2.08	1.04‐4.17	0.038
	≥30	2.37	1.17‐4.80	0.016
Sex	Female			
	Male	1.21	0.76‐1.93	0.423
Neuter status	Entire			
	Neutered	3.30	1.95‐5.59	<0.001
Insurance	Uninsured			
	Insured	3.96	2.42‐6.45	<0.001
Breed	Crossbreed	Baseline	–	–
	Purebreed other	1.24	0.65‐2.37	0.542
	Border collie	1.62	0.37‐7.20	0.524
	Cocker spaniel	2.25	0.73‐6.90	0.157
	Jack Russell terrier	1.12	0.32‐3.92	0.863
	Labradoodle	9.52	3.10‐29.26	<0.001
	Labrador retriever	0.90	0.26‐3.16	0.869
	Standard poodle	51.56	14.60‐182.14	<0.001
	West Highland white terrier	5.87	2.23‐15.46	<0.001

^1^ Wald P‐value

In the final multivariable binary logistic regression model, neuter status, breed, age and insurance status were associated with hypoadrenocorticism (Table 4). Multivariable modelling showed no evidence that sex was associated with hypoadrenocorticism, with males having 1.19 times the odds compared to females (95% CI: 0.74‐1.89; P = 0.471). Neutered dogs had greater odds of hypoadrenocorticism compared to entire dogs (OR: 2.50, 95% CI: 1.44‐4.34, P = 0.001). Breeds with increased odds of hypoadrenocorticism compared to crossbreeds included: standard poodle (OR: 51.38, 95% CI: 14.49‐182.18, P < 0.001), labradoodles (OR: 7.40, 95% CI: 2.40‐22.80, P < 0.001) and the West Highland white terrier (OR: 5.84, 95% CI: 2.20‐15.47, P < 0.001). Dogs aged 4 to <9 years had around twice the odds of hypoadrenocorticism compared to dogs <4 years (OR: 2.07, 95% CI: 1.21‐3.54, P = 0.008). Insured dogs had increased odds of being diagnosed with hypoadrenocorticism in primary‐care practice compared to those that were un‐insured dogs (OR: 3.06, 95% CI: 1.86‐5.04, P < 0.001). The final model considering all cases included the 886 veterinary clinics attended as a random effect, with a small amount of variation in dogs with hypoadrenocorticism demonstrated between veterinary clinics (LRT test of rho P = 0.065, rho: 0.13). The final model demonstrated acceptable model fit (Hosmer lemeshow; P = 0.27) and model discrimination (area under the ROC: 0.78).

**Table 4:** Multivariable logistic regression analysis to assess the risk factors for hypoadrenocorticism in dogs under primary veterinary care in the UK in 2016, for laboratory‐confirmed cases only. Veterinary clinic included as a random effect (laboratory‐confirmed cases n = 72; non‐cases n = 883,436)

Variable	Odds ratio	95% Confidence interval	P‐value^1^
Sex			
Female	Baseline	–	–
Male	1.19	0.74‐1.89	0.471
Neuter status			
Entire	Baseline	–	–
Neutered	2.50	1.44‐4.34	0.001
Breed			
Crossbreed	Baseline	–	–
Purebreed other	1.29	0.67‐2.47	0.436
Border collie	1.72	0.39‐7.64	0.475
Cocker spaniel	2.04	0.66‐6.26	0.214
Jack Russell terrier	1.19	0.34‐4.19	0.788
Labradoodle	7.40	2.40‐22.80	<0.001
Labrador retriever	0.82	0.23‐2.89	0.761
Standard poodle	51.38	14.49‐182.18	<0.001
West Highland white terrier	5.84	2.20‐15.47	<0.001
Age (years)			
<4	Baseline	–	–
4‐ < 9	2.07	1.21‐3.54	0.008
≥9	0.44	0.17‐1.11	0.083
Insurance status			
Uninsured	Baseline	–	–
Insured	3.06	1.86‐5.04	<0.001

^1^ Wald P‐value

## DISCUSSION

This is the largest study to date that reports the frequency, clinical management and risk factors for hypoadrenocorticism in dogs under primary veterinary care in the UK. Hypoadrenocorticism in dogs is not a common diagnosis within UK primary‐care practice; the prevalence of 0.06% reported here is similar to previous estimates in different populations (Hanson *et al*. 2016). In the current study, the annual incidence risk in dogs under primary veterinary care in 2016 was estimated at 0.01% (95% CI: 0.01‐0.02). This is likely a conservative estimate due to unknown dates of diagnoses in 24 (13.6%) dogs which could include some cases diagnosed within 2016.

Access to the free text clinical records within VetCompass allowed examination of the clinical decision‐making within primary‐care practice. This is novel for hypoadrenocorticism because much of the published literature for this disease focuses on cases attending hospital or referral practices (Peterson *et al*. 1996, Kintzer & Peterson 1997, Baumstark *et al*. 2014, Wakayama *et al*. 2017). Seven dogs with a recorded diagnosis of hypoadrenocorticism were excluded from analysis as they had received a “recent” prescription of glucocorticoids before undertaking the ACTH stimulation test; there is evidence that even topical administrations of glucocorticoids can affect responses to ACTH administration (Aniya & Griffin 2008). Therefore these seven dogs could be inaccurately diagnosed with naturally occurring hypoadrenocorticism. The data regarding the diagnosis was unknown for a number of the cases of hypoadrenocorticism due to the realities of using primary‐care practice data for research, with a number of dogs being initially diagnosed at other veterinary clinics (including emergency and referral centres). Dogs without a recorded diagnostic ACTH stimulation test were considered “presumed” cases of hypoadrenocorticism (n = 105/177; 59.3%). Four of the presumed cases had evidence that a basal cortisol concentration had been measured however it is unknown whether an ACTH stimulation test was performed. However, these points highlight the importance of examining primary‐care EPRs for benchmarking purposes and assisting with clinical audit processes (RCVS Knowledge 2020).

The definition and prevalence of “atypical” hypoadrenocorticism is currently contentious (Baumstark *et al*. 2014, Wakayama *et al*. 2017). Due to varying definitions of “atypical” hypoadrenocorticism, prevalence estimates range from 2 to 30% (Melian & Peterson 1996, Peterson *et al*. 1996, Thompson *et al*. 2007, Adamantos & Boag 2008). Within the current population, 10 of 177 (5.6%) cases were recorded as “atypical” by the attending veterinarian, with 3 of 10 dogs having had endogenous aldosterone concentration measured. Due to a lack of consensus regarding its definition, it is likely that differing diagnostic criteria were used by primary‐care veterinarians and there is the possibility that some of the “atypical” cases within this population were false positives.

A high proportion of dogs with a laboratory‐confirmed diagnosis had evidence of hyperkalaemia (88.7%) and hyponatraemia (86.8%) which is comparable to reports in the literature (Peterson *et al*. 1996, Seth *et al*. 2011). The most commonly presenting clinical signs included lethargy, anorexia and weakness, and are largely consistent with previous reports. Vomiting and diarrhoea appeared to be underrepresented in our study compared with reports of higher proportions of cases showing these signs within referral practices (Peterson *et al*. 1996, Seth *et al*. 2011). Information on the presenting clinical signs was not available for dogs that were first diagnosed at referral, emergency or another veterinary clinic. These dogs could have presented differently, possibly with a higher presentation of gastrointestinal signs. IVFT was administered in the majority of dogs (55/72; 76.4%) following a diagnosis suggesting hospitalisation and stabilisation of hypovolaemia was required. Multiple medical maintenance protocols for hypoadrenocorticism were described, with DOCP and prednisolone being the most common (49/72, 68.1%). In March 2016, a newly licensed injectable DOCP was launched in the UK (Dechra Veterinary Products 2016). This resulted in a number of dogs moving from oral fludrocortisone to injectable DOCP. Therefore, the proportional use of each treatment will likely have altered during this time.

The current study found no association between sex and hypoadrenocorticism. Male dogs had 1.19 the odds of hypoadrenocorticism compared to females (95% CI: 0.74‐1.89). There are some similar findings in the literature (Oberbauer *et al*. 2002, Famula *et al*. 2003). However this is contradicted in other studies that reported female dogs with increased risk of hypoadrenocorticism (Peterson *et al*. 1996, Hanson *et al*. 2016), mirroring the sex association observed in human medicine (Erichsen *et al*. 2009, Meyer *et al*. 2014). Neutered dogs had 2.50 times the odds of hypoadrenocorticism compared to entire dogs (95% CI: 1.44‐4.34), with no interactions observed with sex. The reason for the association with neuter status is not known. Although there could be a true association of neutering with hypoadrenocorticism, the observed association could be a proxy measurement for something else such as different demographics of dog ownership, with owners of dogs advocating neutering more likely to attend a veterinary practice and obtain a diagnosis for hypoadrenocorticism. Additionally, the neuter status of cases used for analysis was that recorded within the EPRs at the end of the study period therefore this could differ from the neuter status at the point of diagnosis for some cases.

The current study identified certain breeds with strong associations with hypoadrenocorticism. Previous reports of breed predispositions and inheritance studies have inferred a genetic basis for hypoadrenocorticism (Peterson *et al*. 1996, Oberbauer *et al*. 2002, Famula *et al*. 2003, Hanson *et al*. 2016). The standard poodle had the greatest odds of hypoadrenocorticism compared to crossbreed dogs (OR: 51.38). Labradoodles also had increased odds of hypoadrenocorticism (OR: 7.40). The residual risk for this “designer” breed compared to its predisposed parental breed (standard poodle) suggests that planned cross‐breeding retains some increased risk of disease carried through the standard poodle line, despite claims of hybrid vigour effects in dogs (Nicholas *et al*. 2016). The current study also identified a lack of an association of hypoadrenocorticism with miniature, toy poodles or their crosses, which is supported by the literature, frequently citing the standard as the poodle variant at most risk (Peterson *et al*. 1996, Famula *et al*. 2003, Hanson *et al*. 2016).

The median age at first diagnosis was 5.18 years (IQR: 3.15‐6.79), which is consistent with reports in the literature (Peterson *et al*. 1996, Feldman & Nelson 2004). Dogs aged from 4 to <9 years of age had 2.07 the odds of diagnosis with hypoadrenocorticism compared to those <4 years (95% CI: 1.21‐3.54). Age was non‐linearly associated with a diagnosis of hypoadrenocorticism therefore was categorised for risk factor analysis. When assessed graphically, the non‐linearity indicates that the odds of diagnosis increase rapidly up to around 5 years of age (Fig 1).

Insured dogs had three times the odds of diagnosis with hypoadrenocorticism in primary‐care practice compared to uninsured dogs. This could be because access to the financial security of pet insurance may make reaching a diagnosis more likely (Egenvall *et al*. 2009). Increased odds of hypoadrenocorticism in the insured population suggests that there many uninsured dogs with hypoadrenocorticism not identified in the current study. Such misclassification could explain the slightly higher prevalence of hypoadrenocorticism observed within the Swedish dog population, with a societal culture of high insurance uptake (Hanson *et al*. 2016).

There are some limitations to this study. In particular, the rare outcome examined meant that relatively few cases were recorded, even within this large study population. For this reason, the risk factor analysis included both pre‐existing and incident cases of hypoadrenocorticism in 2016, to increase the power of this study. Inclusion of pre‐existing cases into the risk factor analyses is likely to have introduced some survival bias and therefore the multivariable results should be interpreted as the risk for having, rather than developing, hypoadrenocorticism (O'Neill *et al*. 2020). There were 13.6% of cases that were diagnosed at other veterinary practices (often at emergency or referral practices), resulting in some missing information relating to their diagnosis within this study. The inclusion of the presumed cases could have resulted in some misclassification bias, with dogs incorrectly diagnosed as having hypoadrenocorticism therefore these were excluded from risk factor analysis. Additionally, access to the specific test values from supportive diagnostic ACTH stimulation testing was limited in this study, with most tests performed at external laboratories and not captured within VetCompass. Due to the substantial time requirement for manual verification of cases before inclusion into this study, only 30% of the candidate cases were assessed. Prevalence estimation accounted for this by weighting the unverified candidate cases and a randomised process was used to select those manually verified.

This is the first epidemiological study to provide benchmark data on hypoadrenocorticism in dogs within the UK primary‐care population. About 1 in 1500 UK dogs are affected with hypoadrenocorticism within primary‐care practice, in comparison to 1 in every 50 standard poodles. The risk factor analysis described breed and sex associations with this disorder. These results provide benchmark data for veterinarians within primary‐care practice and could assist clinicians in clinical audit and improved diagnosis of this disease.

### Conflict of interest

IS is supported at the RVC by an award from Dechra Veterinary Products Ltd. The remaining authors have no conflicts of interest to declare.

### Funding

No funding was provided for this study.

### Authors' contributions

All authors made substantial contributions to conception and design, acquisition and extraction of data, and to analysis and interpretation of the results. All authors were involved in drafting and revising the manuscript and gave final approval of the version to be published. Each author agrees to be accountable for all aspects of the accuracy or integrity of the work.
